# Determination of Moisture Content in Oil Palm Fruits Using a Five-Port Reflectometer

**DOI:** 10.3390/s110404073

**Published:** 2011-04-06

**Authors:** Lee Kim Yee, Zulkifly Abbas, Mohamad Ashry Jusoh, You Kok Yeow, Cheng Ee Meng

**Affiliations:** 1 Department of Electrical and Electronic Engineering, Faculty of Engineering and Science, Universiti Tunku Abdul Rahman, 53300 Setapak, Kuala Lumpur, Malaysia; E-Mail: kylee@utar.edu.my; 2 Department of Physics, Universiti Putra Malaysia, 43400 UPM Serdang, Selangor, Malaysia; E-Mail: ashry_jusoh@hotmail.com; 3 Radio Communication Engineering Department (RaCED), Faculty of Electrical Engineering, Universiti Teknologi Malaysia, 81310 Skudai, Johor, Malaysia; E-Mail: kyyou@fke.utm.my; 4 School of Mechatronic Engineering, University Malaysia Perlis, Ulu Pauh Campus, 02600 Arau, Perlis, Malaysia; E-Mail: emcheng@unimap.edu.my

**Keywords:** five-port reflectometer, coaxial sensor, monopole sensor, graphical programming, moisture, data acquisition

## Abstract

This paper presents the development of a PC-based microwave five-port reflectometer for the determination of moisture content in oil palm fruits. The reflectometer was designed to measure both the magnitude and phase of the reflection coefficient of any passive microwave device. The stand-alone reflectometer consists of a PC, a microwave source, diode detectors and an analog to digital converter. All the measurement and data acquisition were done using Agilent VEE graphical programming software. The relectometer can be used with any reflection based microwave sensor. In this work, the application of the reflectometer as a useful instrument to determine the moisture content in oil palm fruits using monopole and coaxial sensors was demonstrated. Calibration equations between reflection coefficients and moisture content have been established for both sensors. The equation based on phase measurement of monopole sensor was found to be accurate within 5% in predicting moisture content in the fruits when compared to the conventional oven drying method.

## 1. Introduction

The principle of the six-port reflectometer for measuring reflection coefficients was introduced by Engen [1] in 1977. Since then, many papers have been published to simplify or extend the application of the instrument [2–6]. An interesting variant of the six-port unit is provided by the five-port reflectometer where the reflection coefficient of the device under testing can be determined by using only three power detectors [5,6].

Previously, we proposed the use of an open ended coaxial sensor to determine moisture content in oil palm fruits [7] by measuring the reflection coefficient with a Vector Network Analyzer (VNA). In this paper, we present an extension of the technique by measuring the reflection coefficient of the fruits using both open ended coaxial and monopole sensors with a five port reflectometer without requiring the use of VNA.

## 2. Five Port Reflectometer

### 2.1. Measurement Setup

**Figure 1.**
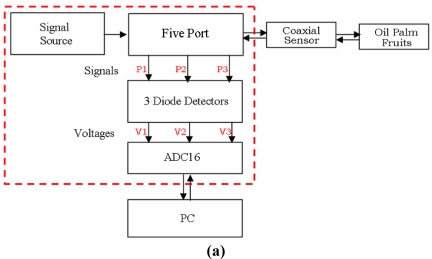

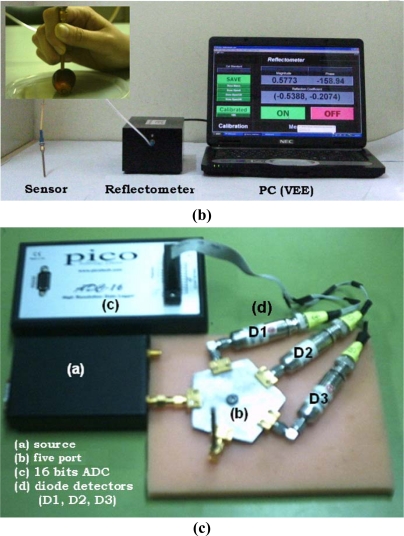
Five-port reflectometer. **(a)** five-port reflectometer measurement setup; **(b)** photo of five-port reflectometer; **(c)** five-port reflectometer main components.

The measurement setup, shown in Figure 1, consists of a PC, five-port circuit, a 16 bits Analog/Digital converter PICO ADC16 and a continuous wave signal source (NovaSource M2 NS2-1700104) with a constant 10 dBm output power at 2 GHz. The signals reflected from the sensor are measured using diode detectors (D1, D2, D3) at each output port P1, P2 and P3. The detectors were MIDISCO MDC1087-S Zero Bias Schottky diodes having ±0.5 dB flatness, max VSWR 1.2:1 with an SMA input and a BNC output. The sensors used in this work were the open ended coaxial and monopole sensor [7,8] constructed from an RG 402 semi-rigid cable.

### 2.2. The Five-Port Circuit Configuration

**Figure 2.**
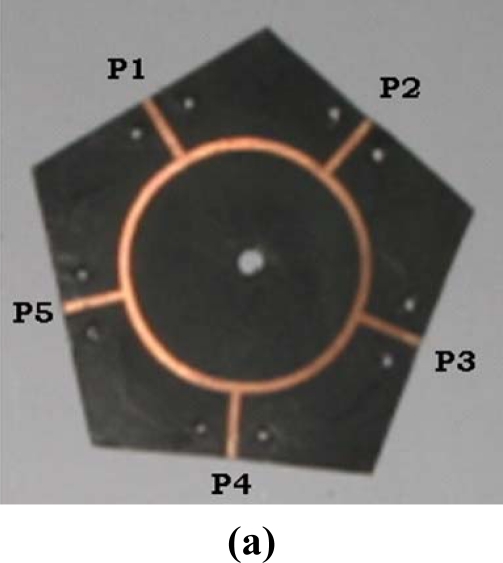

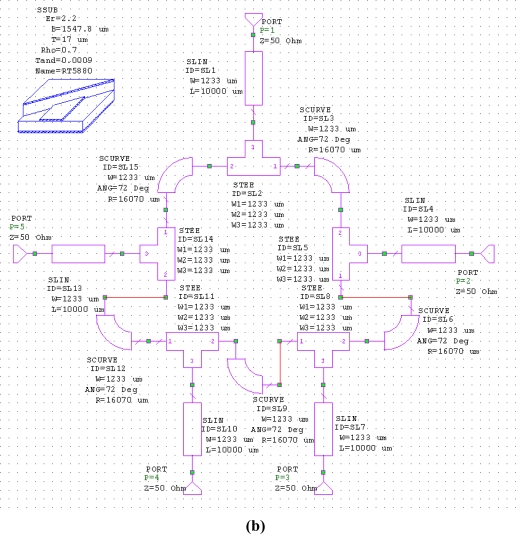
Five-port circuit. **(a)** Fabricated circuit; **(b)** Microwave Office Workspace.

The five-port circuit shown in Figure 2 was designed at 2 GHz using Microwave Office software version 4.3. The permittivity and thickness of the substrate were ɛ_r_ = 2.2 and 1.5748 mm, respectively.

The widths of each arm and ring of the five-port were *w*_1_ = 1.008 mm and *w*_2_ = 1.272 mm. The radius of the ring was *r* = 16.968 mm. The components *SLIN*, *SSUB*, *STEE* and *SCURVE* in Figure 1(b) represent the microstrip line, substrate, T junction and bend, respectively.

### 2.3. Calibration Procedure

The relationship between the complex reflection coefficient Γ of the unknown load and the power ratios w_i_ can be written in the form [5,6]:

(1) $${\left|\Gamma -q_{i}\right|}^{2}={\left|w_{i}\right|}^{2}$$

where:

(2) Γ=x+jy

(3) $$q_{i}=u_{i}+{\text{jv}}_{i}$$

(4) $${\left|w_{i}\right|}^{2}=P_{i}/k_{i}$$

with i = 1, 2 and 3. P_i_ are the powers measured by the three detectors, k_i_ are the unknown constants to be determined from the calibration procedure and q_i_ are the values of the calibration standards. The four calibration standards used in this work to determine the unknown constant k_i_, x_i_, and y_i_ were a precision 50 Ω load, standard open standard, offset 120° and offset 240° open calibration standards. Assuming perfect matched load and open standards, we obtained from Equations (1–4):

(5) $$\frac{P_{i}}{k_{i}}=u_{i}^{2}-{2u}_{i}x_{i}+x_{i}^{2}+v_{i}^{2}-{2v}_{i}y_{i}+y_{i}^{2}$$

At port 1 (I = 1):

(6) $$\frac{P_{1}}{k_{1}}=u_{1}^{2}-{2u}_{1}x_{1}+x_{1}^{2}+v_{1}^{2}-{2v}_{1}y_{1}+y_{1}^{2}$$

After some algebraic manipulation of Equation (6), the unknown values k_1_, x_1_, and y_1_ for port 1 can be determined from the linear equations system of Equation (7) by measuring the the four calibration standards:

(7) $$\left(\begin{array}{ccc} P_{1\text{open}(0)}-P_{1m} & 2\left(u_{1\text{open}(0)}-u_{1m}\right) & 2\left(v_{1\text{open}(0)}-v_{1m}\right)\\ P_{1\text{open}(120)}-P_{1m} & 2\left(u_{1\text{open}(120)}-u_{1m}\right) & 2\left(v_{1\text{open}(120)}-v_{1m}\right)\\ P_{1\text{open}(240)}-P_{1m} & 2\left(u_{1\text{open}(240)}-u_{1m}\right) & 2\left(v_{1\text{open}(240)}-v_{1m}\right) \end{array}\right)\left(\begin{array}{c} \frac{1}{k_{1}}\\ x_{1}\\ y_{1} \end{array}\right)=\left(\begin{array}{c} u_{1\text{open}(0)}^{2}+v_{1\text{open}(0)}^{2}-u_{1m}^{2}-v_{1m}^{2}\\ u_{1\text{open}(120)}^{2}+v_{1\text{open}(120)}^{2}-u_{1m}^{2}-v_{1m}^{2}\\ u_{1\text{open}(240)}^{2}+v_{1\text{open}(240)}^{2}-u_{1m}^{2}-v_{1m}^{2} \end{array}\right)$$

The same procedure can similarly be used to determine other k_i_, x_i_, and y_i_ values for ports 2 and 3.

### 2.4. Determination of the Reflection Coefficient of Device under Test

From Equation (5), the power ratios at port 1, 2 and 3 can be written in the form:

(8) $$\frac{P_{1}}{k_{1}}=u_{1}^{2}-2u_{1}x_{1}+x_{1}^{2}+v_{1}^{2}-{2v}_{1}y_{1}+y_{1}^{2}$$

(9) $$\frac{P_{2}}{k_{2}}=u_{2}^{2}-{2u}_{2}x_{2}+x_{2}^{2}+v_{2}^{2}-{2v}_{2}y_{2}+y_{2}^{2}$$

(10) $$\frac{P_{3}}{k_{3}}=u_{3}^{2}-{2u}_{3}x_{3}+x_{3}^{2}+v_{3}^{2}-{2v}_{3}y_{3}+y_{3}^{2}$$

For the device under test, u_1_ = u_2_ = u_3_ = u and v_1_ = v_2_ = v_3_ = v can be obtained from the following matrix:

(11) $$\left(\begin{array}{cc} -{2x}_{1}+{2x}_{2} & -{2y}_{1}+{2y}_{2}\\ -{2x}_{2}+{2x}_{3} & -{2y}_{2}+{2y}_{3} \end{array}\right)\left(\begin{array}{c} u\\ v \end{array}\right)=\left(\begin{array}{c} \frac{P_{1}}{k_{1}}-\frac{P_{2}}{k_{2}}-x_{1}^{2}+x_{2}^{2}-y_{1}^{2}+y_{2}^{2}\\ \frac{P_{2}}{k_{2}}-\frac{P_{3}}{k_{3}}-x_{2}^{2}+x_{3}^{2}-y_{2}^{2}+y_{3}^{2} \end{array}\right)$$

**Figure 3.**
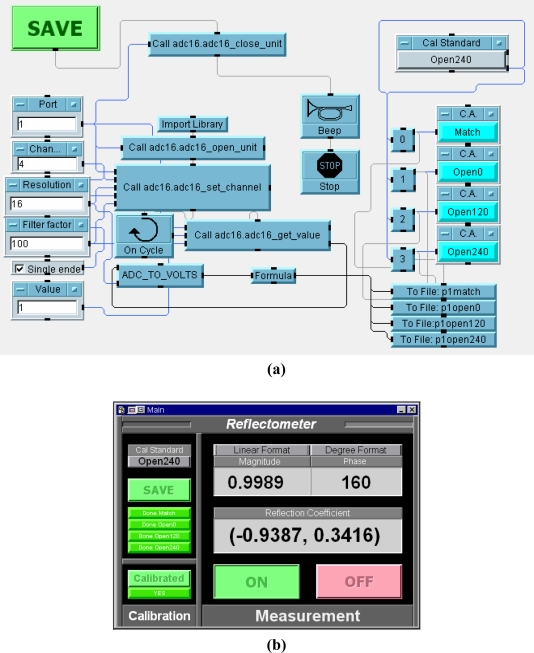
Five-port reflectometer software. **(a)** Data acquisition module; **(b)** Panel view.

A computer program has been developed to control, acquire, and save data using the Agilent VEE version 7.0 graphical programming software. The program was also used to implement all the calibrations and calculations of the reflection coefficients. As an example, the ADC 16 data acquisition module is illustrated in Figure 3(a). In the final form, all the VEE objects are hidden and only the panel view of the Five-Port Reflectometer in run-time mode as shown in Figure 3(b) is accessible to the end user.

## 3. Results and Discussion

### 3.1. Reflectometer Performance

The performance of the five-port reflectometer was tested by comparing reflection coefficient S_11_ values of eight different offset shorts using both the reflectometer and a commercial VNA. The results are listed in Table 1.

The mean error in magnitude between the reflectometer and VNA measurements was 0.0202, whilst the phase mean error was 1.91°. The accuracy of the reflectometer was further tested by comparing the reflection coefficient S_11_ measurement results between the reflectometer and the VNA for several well known materials using both the monopole and open ended coaxial sensors. Again good agreement between the reflectometer and VNA results were obtained for all the materials listed in Tables 2 and 3 for the monopole and open ended coaxial sensors, respectively. The maximum errors between the VNA and reflectometer when using the open ended coaxial sensor were 0.0318 and 3.89° (or 3.18% and 1.08%) for magnitude and phase, respectively. The corresponding errors were 0.0304 and 3.14° (or 3.04% and 0.87%) for the monopole sensor.

### 3.2. Determination of Moisture Content in Oil Palm Fruits

More than 100 fruits in various stages of fruit ripeness were measured using both the coaxial and monopole sensors in conjunction with the five-port reflectometer. Abnormal, not fully developed, dry, and rotten fruits were not considered. The surface of the fruit was wiped dry to free excess surface moisture. All the reflection coefficient S_11_ measurements of the fruit samples using the five-port reflectometer were done at 26 °C. The samples were then dried in a forced-air oven for four days at 105 °C for moisture content determination on a wet basis [7].

**Figure 4.**
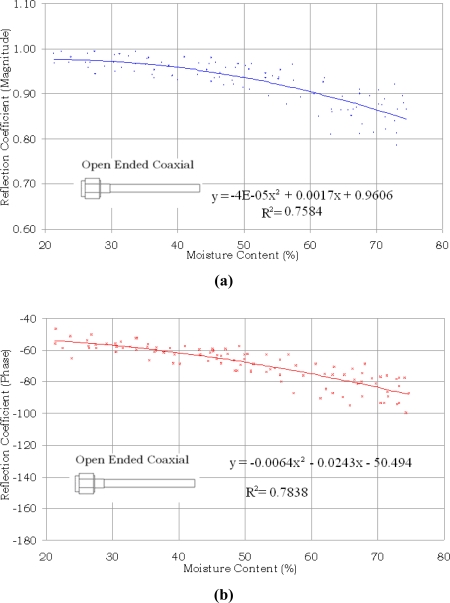
Variation in magnitude and phase of S_11_ with moisture content using open ended coaxial sensor. **(a)** Magnitude; **(b)** Phase.

**Figure 5.**
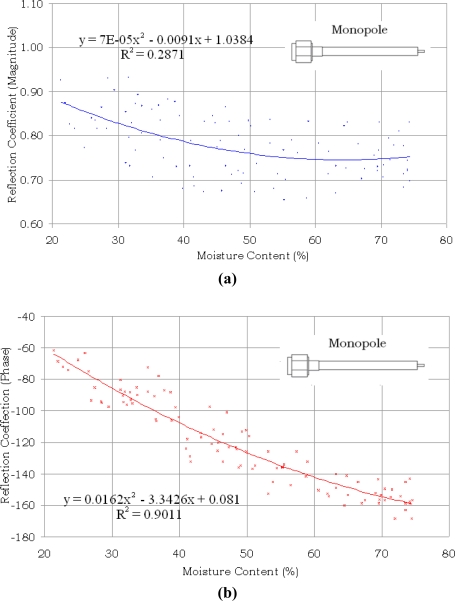
Variation in magnitude and phase of S_11_ with moisture content using monopole sensor. **(a)** Magnitude; **(b)** Phase.

The variations in the magnitude and phase of S_11_ with moisture content in oil palm fruits are shown in Figures 4 and 5 for the open ended coaxial and monopole sensors, respectively. It can be clearly seen from Figure 4 that both magnitude and phase of the reflection coefficient generally decrease with increasing moisture content in the range between 21% and 75% for the open ended coaxial probe.

However, for the monopole sensor shown in Figure 5, only the phase showed a tendency to decrease with increasing moisture content, whilst the magnitude values were randomly scattered with variation in moisture content. The equation and the determination coefficient R^2^ shown in each graph were obtained by applying regression analysis. The determination coefficients for both magnitude and phase methods of the open ended coaxial sensor were almost similar. As expected, the highest correlation was obtained from phase measurements using monopole sensor. The phase of S_11_ is highly sensitive not only to the length of the extended the inner conductor of the monopole sensor but also sensitive to small variation in the complex permittivity of the samples due to different percentages of moisture content. The weak correlation for the magnitude measurement using monopole sensor was due to multiple wave reflection between the extended inner conductor and fruit.

**Figure 6.**
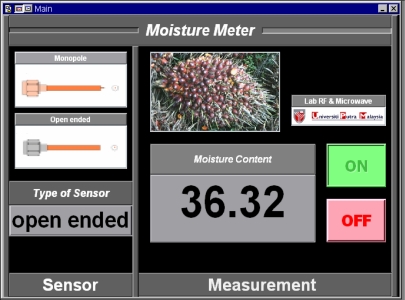
Panel View of VEE Program to determine moisture content in oil palm fruits.

A computer program was developed using the Agilent VEE software to predict moisture content in oil palm fruits by applying inverse relationships to the equations in Figures 4 and 5. A panel view of the program is illustrated in Figure 6.

**Figure 7.**
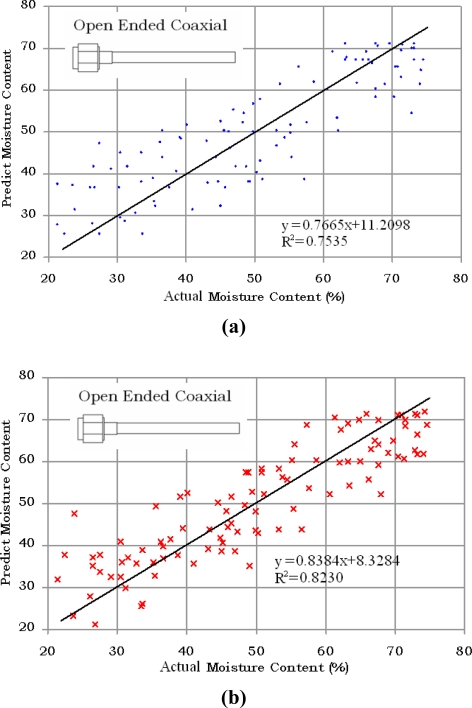
Comparison between predicted and actual moisture content. **(a)** Magnitude; **(b)** Phase methods using coaxial sensor.

**Figure 8.**
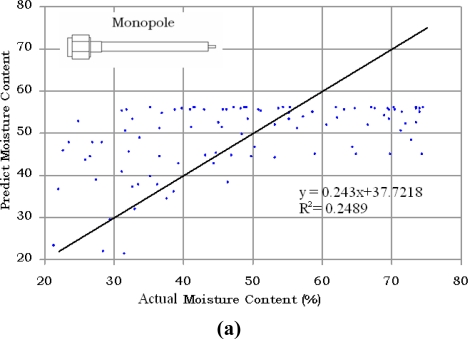

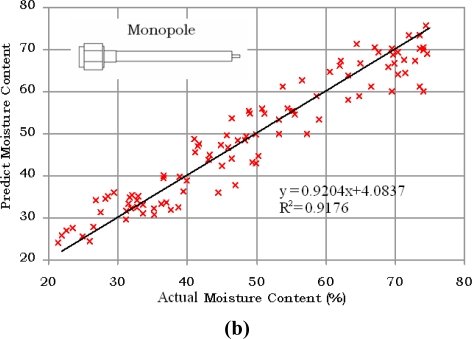
Comparison between predicted and actual moisture content. **(a)** Magnitude; **(b)** Phase methods using monopole sensor.

The results for a different batch of 100 samples of oil palm fruits using open ended coaxial and monopole sensors are compared with the actual values of moisture content obtained using standard oven drying method in Figures 7 and 8, respectively. The phase measurement of the monopole is the most accurate technique to predict the moisture content in oil palm fruits. In contrast, the magnitude of monopole is saturated and has a complex relationship with moisture content. The strong saturation effect in Figure 8(a) was due to fringing fields created by the interaction between the monopole back lobe radiation and the sample. A ground-plane flange is required to minimize the influence of this external noise and can also be used to increase the directivity of the monopole sensor. However, because of its size, a ground-plane flange monopole sensor is not suitable for small samples such as the oil palm fruits. The mean absolute errors were 6.78% and 5.95% when using the magnitude and phase equations of the open ended coaxial sensor, respectively. The lowest absolute mean error was 3.73% when using phase method with the monopole sensor. However, as expected from Figure 5(a), the monopole sensor also recorded the highest absolute mean error (11.32%) when using the magnitude method. This was due to multiple reflection along the interface between the extended inner conductor and fruit.

## 4. Conclusions

The five-port reflectometer represents a simple, cheap and efficient microwave network analyzer solution to determine reflection coefficients which in turn can be used directly to determine the moisture content in fruits. The reflectometer is especially useful for *in-situ* determination of the quality of fruits in the field. The detailed calibration procedure has been described. The five port reflectometer circuit was designed and tested against several known standards. The measured reflection coefficient results were in good agreement with the results obtained using a commercial network analyzer. A computer program has also been developed to perform the calibration and calculate the reflection coefficients, which in turn were used to determine moisture content in the oil palm fruits. A prototype of a general five-port reflectometer has been developed and tested for the determination of moisture content in oil palm fruits by using monopole and coaxial sensors. The predicted moisture content based on magnitude and phase measurements of the two sensors were compared with actual moisture content obtained from a standard oven drying method. The phase method of the monopole sensor showed the highest accuracy, with a mean absolute error 3.73%.

## Figures and Tables

**Table 1.** Magnitude and phase of reflection coefficient measured using reflectometer and commercial VNA.

**Standard**	**VNA**		**Reflectometer**		**Deviation**	
	**Magnitude**	**Phase**	**Magnitude**	**Phase**	**Magnitude**	**Phase**
**Std 1**	0.9860	−30.30	0.9931	−31.22	0.0071	0.92
**Std 2**	0.9990	−69.61	1.0070	−72.13	0.0080	2.52
**Std 3**	0.9860	−138.93	1.0210	137.40	0.0350	1.53
**Std 4**	1.0042	−105.50	1.0200	107.20	0.0158	1.70
**Std 5**	1.0030	180.00	1.0410	177.10	0.0380	2.90
**Std 6**	0.9950	129.50	0.9850	131.40	0.0100	1.90
**Std 7**	0.9910	52.60	0.9750	50.20	0.0660	2.40
**Std 8**	0.0200	9.67	0.0027	8.25	0.0173	1.42
			**Mean**		**0.0202**	**1.91**

**Table 2.** S_11_ of several known materials using monopole sensor.

	**VNA**		**Reflectometer**	
	**Magnitude**	**Phase**	**Magnitude**	**Phase**
**Air**	0.9845	−30.00	0.9637	−33.72
**Water**	0.9050	−173.53	0.8980	−169.77
**Ethanol**	0.4712	−80.53	0.5078	−81.96
**Propanol**	0.7257	−49.56	0.6939	−52.15
**Methanol**	0.6773	−154.56	0.7040	−150.67

**Table 3.** S_11_ of several known sample using open ended coaxial.

	**VNA**		**Reflectometer**	
	**Magnitude**	**Phase**	**Magnitude**	**Phase**
**Air**	0.9829	138.53	0.9582	141.29
**Water**	0.8841	57.55	0.9143	58.50
**Ethanol**	0.8091	127.24	0.8093	130.04
**Propanol**	0.7403	90.51	0.7701	90.40
**Methanol**	0.8939	133.08	0.8890	136.22
